# Peripheral mechanisms of neuropathic pain – involvement of lysophosphatidic acid receptor-mediated demyelination

**DOI:** 10.1186/1744-8069-4-11

**Published:** 2008-04-01

**Authors:** Hiroshi Ueda

**Affiliations:** 1 Division of Molecular Pharmacology and Neuroscience, Nagasaki University Graduate School of Biomedical Sciences, 1-14 Bunkyo-machi, Nagasaki 852-8521, Japan

## Abstract

Recent advances in pain research provide a clear picture for the molecular mechanisms of acute pain; substantial information concerning plasticity that occurs during neuropathic pain has also become available. The peripheral mechanisms responsible for neuropathic pain are found in the altered gene/protein expression of primary sensory neurons. With damage to peripheral sensory fibers, a variety of changes in pain-related gene expression take place in dorsal root ganglion neurons. These changes, or plasticity, might underlie unique neuropathic pain-specific phenotype modifications – decreased unmyelinated-fiber functions, but increased myelinated A-fiber functions. Another characteristic change is observed in allodynia, the functional change of tactile to nociceptive perception. Throughout a series of studies, using novel nociceptive tests to characterize sensory-fiber or pain modality-specific nociceptive behaviors, it was demonstrated that communication between innocuous and noxious sensory fibers might play a role in allodynia mechanisms. Because neuropathic pain in peripheral and central demyelinating diseases develops as a result of aberrant myelination in experimental animals, demyelination seems to be a key mechanism of plasticity in neuropathic pain. More recently, we discovered that lysophosphatidic acid receptor activation initiates neuropathic pain, as well as possible peripheral mechanims of demyelination after nerve injury. These results lead to further hypotheses of physical communication between innocuous Aβ- and noxious C- or Aδ-fibers to influence the molecular mechanisms of allodynia.

## Background

Chronic pain should be considered to be a disease rather than just a symptom, because it is one of most common reasons for hospital visits. Although recent advances in molecular biology techniques, and the subsequent discoveries of key molecules involved in pain production, have clearly contributed to better understanding acute pain [1-4], the molecular mechanisms underlying chronic pain remain to be fully clarified.

Chronic pain, or specifically neuropathic pain, is quite different from other types of pain, such as nociceptive (or physiological) or inflammatory pain, because it is irreversible, even when the underlying cause has been rectified [2]. For this reason, the proper diagnosis and early treatment are often difficult. Moreover, neuropathic pain commonly occurs as a secondary symptom in diseases, such as diabetes, cancer, and herpes zoster infection, or as a side effect of chemotherapeutic treatments [3,5-7]. Neuropathic pain is often characterized by stimulus-independent persistent pain or abnormal sensory perception of pain, such as allodynia (pain perception upon the innocuous tactile stimuli) and hyperalgesia (exaggerated pain sensations by mildly noxious stimuli) [3,8]. To treat chronic pain, we must first understand the initial and sustained molecular events in experimental animal models. Because the central mechanisms of sustained molecular events, which are closely related to memory in the brain, have been described in elsewhere in detail [9,10], this review focuses on peripheral mechanisms of initial events from nociceptors to the spinal dorsal horn.

### Approaches to study plasticity in nociceptor endings

Neuropathic pain occurs as a consequence of complex sensory dysfunction and may differ depending on the type of insult and the individual patient. Furthermore, due to the dynamic nature of the pain system, signs and symptoms of neuropathic pain change over time. Injury to peripheral nerves causes functional and biochemical changes at the site of injury, as well as to other areas of the affected nerve, and later to higher order neurons in the spinal cord and brain [3,8-12]. Nociceptor endings cause a generator potential, which leads to an action potential in polymodal C and mechanothermal Aδ fibers [1,13]. These action potentials are then conducted to higher centers in the central nervous system (CNS) via neurotransmitter release and are accompanied by a variety of responses, including withdrawal reflexes, conscious perception of pain, and emotional effects. The pain signal, on the other hand, drives the descending noradrenergic and serotonergic pain-inhibitory systems from the lower brain stem to the spinal dorsal horn [14]. Therefore, chronic neuropathic pain is a result of problems in ascending pain transmission or descending pain-inhibitory system.

The identification of mechanisms or key molecules related to hyperalgesia and allodynia in neuropathic pain could be elucidated by studies using antisense oligos, RNA interference (RNAi), or transgenic (KO) mice lacking specific genes. However, these approaches present difficulties, such as: 1) intrathecal treatments with antisense oligo or RNAi cannot specify whether the action site is on sensory fibers or the spinal cord, although some studies have demonstrated dorsal root ganglion (DRG)-specific down-regulation [15,16]; 2) the availability of specific KO mice is limited, and functional compensation during development and growth may modify the roles of the genes involved; and 3) the availability of conditional KO mice is even more limited. Considering this, as an initial attempt to survey key molecules responsible for neuropathic pain, we have developed novel nociception tests to measure algogenic-induced paw flexion (APF) [3].

### Pharmacological plasticity in neuropathic hyperalgesia

Our laboratory has developed a highly sensitive and minimally stressful nociception test (APF test) in mice [3]. In this test, the mouse is held in a hammock-type cloth sling, which is suspended from a bar, and nociceptive responses (in force) that are induced by intraplantar injection of a receptor ligand or algogenic substance can be measured. The APF test has proven to be advantageous for the study of *in vivo *pain signal transduction at peripheral nerve endings and has enabled the characterization of pain induction through nociceptors on C- or Aδ-fibers. *In vivo *signal transduction can be studied through the use of pharmacological methods using intraplantar treatments with various inhibitors and intrathecal pretreatments with antisense oligodeoxynucleotide (AS-ODN). The AS-ODN treatments are intended to determine whether the action site of compounds (i.pl.) reside on nociceptor endings or on non-neuronal peripheral cells.

Pharmacological studies have shown that the nociceptive fibers are responsible for various algogenics actions, which can be divided into three groups [17]. Neonatal pretreatment of capsaicin degenerates unmyelinated C fibers, polymodal nociceptive fibers [18-20]. Spinal antagonism, using receptor antagonists for substance P (SP) or glutamate, is another way to characterize nociceptive fibers. The use of neonatal capsaicin-sensitivity and spinal antagonism enabled us to pharmacologically categorize algogenic-induced nociceptive flexor responses into three groups [17]: 1) subcutaneous (s.c.) capsaicin-pretreatment and/or intrathecal (i.t.) pretreatment with NK1-type SP receptor antagonist abolished nociceptive responses to bradykinin (BK), SP, and histamine (His), which are representative chemical mediators; 2) ATP-induced responses were abolished with pretreatments of neonatal capsaicin (s.c.) or NMDA receptor antagonist (i.t.), but not by NK1 antagonist (i.t.); 3) PGI_2 _receptor agonist-induced responses were not affected by neonatal capsaicin, but were abolished by the NMDA antagonist. Therefore, we proposed that C-fibers could be further divided into two groups: peptidergic type 1 (SP-containing) and non-peptidergic type 2 (P2X_3 _receptor-expressing) C-fibers. This proposal is similar to the schematic model proposed by Snider & McMahon [21], who report two groups of C-fibers with differential gene (or protein) expression. This sub-classification of C-fibers is anatomically supported elsewhere [22]; only 3% of P2X_3 _receptor-positive neurons co-existed with SP in rat DRG neurons. Our proposal suggested a third group (type 3) of nociceptive fibers that may be related to myelinated A(δ) fibers, as opposed to C fibers [3].

Partial injury to peripheral nerves has been used to generate representative experimental animal models for neuropathic pain [23], where remarkable functional and molecular changes are observed. The injury-induced expression of Na_v_1.3 in large (A-fiber) DRG neurons [24] is thought to be one of the mechanisms underlying the hypersensitivity. We have also observed injury-induced appearance of TRPV1 and B1-type BK receptors in N52 (an A-fiber marker)-positive DRG neurons [15,25]. Neuropeptides, such as galanin, neuropeptide Y (NPY), and pituitary adenylyl cyclase activating polypeptide (PACAP), which are normally expressed at low levels in sensory neurons, are dramatically increased in DRG neurons, including medium to large size neurons (A-fibers) [26-29]. Recently, a paper described reduced neuropathic pain in mice lacking PACAP [27]. Nevertheless, the up-regulation of these neuropeptides is not always the mechanism intrinsic to neuropathic pain. NPY-null mice exhibit autotomy-like biting behaviors [30], augmentation of neuropathic pain is observed in NPY Y1 receptor-null mice [31], and i.t. injection of NPY inhibits neuropathic pain [32]. Similarly, neuropathic pain is decreased in transgenic mice overexpressing galanin, and augmented in galanin receptor 1-null mice [33,34]. On the contrary, some molecules, such as substance P [28,35], Na_v_1.8, and Na_v_1.9, are down-regulated [36,37]. These modifications may be generalized functional switches of C-fibers to A-fibers, as evidenced by the down-regulation of C-fiber mechanisms and the up-regulation of A-fiber mechanisms [3].

### Sensory fiber-specific plasticity in neuropathic allodynia

Allodynia is the best target to investigate molecular mechanisms underlying neuropathic pain. In the APF test, hypoalgesia could be characterized through peptidergic C-fibers and A(δ)-hyperalgesia, another feature of neuropathic pain. This test mimics the physiological event where, upon exposure to noxious stimuli, chemicals are secreted in the vicinity of nerve endings to stimulate these nociceptive fibers. However, because the peripheral nerve terminals of innocuous Aβ fibers, which are thought to mediate allodynia, are covered by nonneuronal supporting cells, and protected from chemicals, different nociception tests are needed to characterize Aβ-fibers.

**Figure 1:**
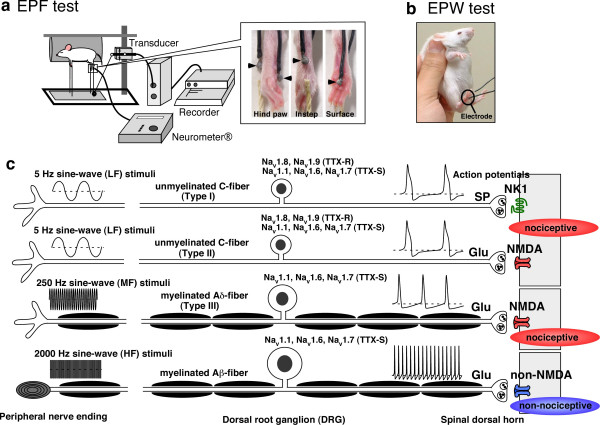
**Schematic model of electric stimulation-induced paw flexion (**a**, EPF) and paw withdrawal (**b**, EPW) test in mice.** (**c) **Frequency-specific stimulation of different sensory fibers is closely related to differential expression of voltage-dependent Na_v _channels, which have distinct kinetics patterns during activation, inactivation, and recovery from inactivation or repriming. Because type I and II C-fibers are stimulated by LF-stimuli, nociceptive responses or p-ERK signals can be blocked by neonatal capsaicin pretreatment, or by NK1 and NMDA receptor anatgonists. Aδ- and Aβ-fibers, on the other hand, are stimulated by MF or HF stimuli, respectively. NMDA or non-NMDA receptor antagonists block spinal transmission caused by MF or HF-stimuli, respectively.

In order to characterize Aβ fiber-mediated pain transmission, we have recently developed novel nociception tests, using the Neurometer^®^, called electrical stimulation-induced paw flexion (EPF) and paw withdrawal (EPW) tests [7,38]. The EPF test uses the same apparatus as the APF test; however, the EPF test utilizes electrodes placed on the plantar surface and instep, rather than a cannula filled with drug solution (Fig. 1a). In the EPW test, the mouse is hand-held and electrical stimulation is applied to the paw (Fig. 1b). Latency of paw withdrawal behavior is evaluated as the nociceptive threshold. In both the EPF and EPW tests, the threshold is consistently reproducible, even after repeated applications to the same mouse. According to the manufacturer's protocol for Neurometer^®^, low (LF, 5 Hz), medium (MF, 250 HZ), and high frequency (HF, 2000 Hz) electrical stimulation results in stimulation of C-, Aδ-, and Aβ-fibers, respectively [39,40]. Through the use of electrophysiology, Koga *et al*. [41] confirmed frequency-specific stimulation of sensory fibers characterization with the Neurometer^®^. This specificity could be attributed to different electrical characteristics of C-, Aδ-, and Aβ-fibers. C-fibers generate a slow sodium-dependent spike due to the presence of tetrodotoxin (TTX) – resistant sodium channels, Na_v_1.8 and Na_v_1.9, which exhibit slow kinetics patterns for activation, inactivation, and recovery from inactivation or repriming, while A-fibers predominantly express TTX-sensitive channels such as Na_v_1.1, Na_v_1.6, Na_v_1.7, which exhibit fast kinetics patterns to allow high frequency firing [42,43], as shown in Fig. 1c.

Matsumoto *et al*. [38] also confirmed frequency-specificity with a pharmacological study; neonatal capsaicin treatments to cause a damage of C-fibers eliminated LF-stimuli-induced nociceptive behavior, but not MF- or HF-stimuli-induced behaviors. These results suggest that nociceptive behavior from LF-stimuli is a result of C-fiber stimulation, while MF- or HF-stimuli-induced behavior is due to A-fiber stimulation. Furthermore, MF-stimulus to the finger results in sharp pain (prickly feeling), while HF-stimulus causes an unpleasant vibrating perception (light tickle).

Further pharmacological characterizations strengthened the validity of behavioral studies using Neurometer^® ^[38,44]. Both NK1 and NMDA receptor antagonists inhibited LF-stimuli-induced nociceptive responses; NMDA receptor antagonists inhibited MF-responses, while AMPA/kainate (non-NMDA) receptor antagonists specifically inhibited HF-induced behaviors (Fig. 1c), which is consistent with previous reports [45]. The characteristics of pain behavior were consistent to those obtained with the measurement of neuron-specific phosphorylation of extracellular signal-regulated kinase 1/2 (p-ERK). Studies [46]have shown that LF-stimuli induced neuronal p-ERK signals at the spinal dorsal horn (lamina I and II) were inhibited by NK1 and NMDA receptor antagonists, while MF-induced signals (lamina I) were specifically inhibited by the NMDA receptor. Moreover, HF stimuli resulted in no signal in the spinal cord. Thus, it seems likely that LF-stimuli causes C-fiber stimulation, while MF- or HF-stimuli causes nociceptive A(δ) or innocuous A(β)-fiber stimulation, respectively, in naïve animals and humans. It is unlikely that cutaneous application of high frequency (250 or 2000 Hz) electrical stimuli is capable of completely stimulating sensory fibers at the same frequency. Koga *et al*. [41] reported ~3 Hz C-fiber, ~22 Hz Aδ-fiber, and ~140 Hz Aβ-fiber responses after 5, 250, and 2000 Hz stimulation, respectively; these responses are similar to physiological levels [47].

### Functional mechanisms influencing neuropathic allodynia

According to the previous hypothesis for neuropathic allodynia, nerve damage results in a retraction of C-fibers and allows for intrusion of A-fibers to the newly created space and loss of C-fiber pain transmission. This hypothesis was originally proposed with anatomical studies that demonstrated that nerve transection produces central sprouting of large fibers from deep laminae to lamina II [48,49]. Although nerve injury-induced intrusion of Aβ-fibers to the superficial dorsal horn layers has not been well documented, this hypothesis received functional support through electrophysiological studies [50].

Behavioral studies (APF test) have determined a loss of peptidergic C-fiber responses after nerve injury [25,35]. The down-regulation of C-fiber specific molecules, such as Na_v_1.8 and 1.9 [36,37] and bradykinin B2 receptor [15], could be responsible for this type of neuropathic pain. The down-regulation of pain transmitter SP in the dorsal horn of spinal cord could be another mechanism [3,35,51]. Because SP precursor preprotachykinin (PPT)-null mice continued to exhibit neuropathic pain, the loss of peptidergic C-fiber function may not be significant stimuli to cause nociceptive behaviors. Alternatively, up-regulation of NK1 receptor expression in the spinal cord [52] may counteract the threshold change.

On the other hand, hypersensitization or allodynia upon innocuous stimulation has been studied. These studies demonstrated, in particular, the up-regulation of large-fiber-specific molecules [15,24,25,27]. Although these changes may be responsible for neuropathic hyperalgesia, they cannot explain the mechanism of allodynia. The novel method using the Neurometer^® ^clearly demonstrates Aβ-fiber stimulation-induced nociceptive flexor responses [38]. Following partial sciatic nerve injury, LF-stimuli-induced behavioral responses were significantly inhibited in the EPF test, while MF-stimuli-induced responses were enhanced [38]. These results are in accordance with studies using the APF test to determine that peptidergic C-fiber responses were lost, while Aδ-fiber responses were hypersensitized [25]. It should be noted that similar injury-specific hypersensitization was also observed with HF-stimuli [38,44], which should stimulate innocuous fibers in naïve mice. Consistent results were also observed when neuronal p-ERK signals were measured at the spinal dorsal horn [46]. Most interestingly, HF-stimuli generated significant p-ERK signals at the spinal cord lamina I and II after nerve injury. Pharmacological characterization after the injury also verified the plasticity that HF-stimuli (Aβ)-induced spinal transmission is mediated by NMDA receptor, but not by non-NMDA receptor [44,46].

Thus, functional changes following nerve injury suggest that two molecular events are taking place: communication between different sensory fibers and C-fiber retraction. Demyelination, and subsequent physical cross-talk, might be the mechanism responsible for neuropathic pain, because many demyelinating diseases accompany chronic pain, as with Guillain-Barre syndrome [53], Charcot-Marie-Tooth type I disease [54], and multiple sclerosis [55]. Indeed, it has been reported that neuropathic pain develops as a result of aberrant myelination (splitting, detachment, and loss of myelin) in mice deficient in the myelin protein periaxin [56]. However, little is known of the molecular events causing demyelination in the diseases that accompany neuropathic pain.

### Lysophosphatidic acid (LPA) mimics nerve injury-induced neuropathic pain

A number of pharmacological studies suggest that lysophosphatidic acid (LPA) might cause neuropathic pain and demyelination following partial sciatic nerve injury. LPA is one of several lipid metabolites released after tissue injury, as well as from various cancer cells [57-59]. LPA receptors activate multiple signaling pathways and multiple G-proteins [60-64]. Direct stimulation of peripheral nociceptor endings by LPA, through LPA_1 _receptors, also suggests a role in nociceptive processes [65,66]. Of particular note, receptor-mediated LPA signaling via Gα_12/13 _activates the small GTPase RhoA [63,64,67]. In the active state, Rho is translocated to the plasma membrane and thereby relays extracellular signals to multiple downstream effectors, including Rho-kinase or ROCK, which can be inhibited by the pyridine derivative compound, Y-27632. Inhibition of the Rho pathway can also be accomplished by selective ADP-ribosylation of RhoA, using Clostridium botulinum C3 exoenzyme (BoTN/C3). The involvement of Rho-ROCK system in neuropathic pain mechanisms was initially demonstrated by i.t. injections of BoTN/C3 prior to peripheral nerve injury in mice, which blocked the development of hyperalgesia [68]. LPA receptors and LPA receptor gene expression activates Rho in peripheral nerves [69-71], which suggests that LPA receptors might pathophysiologically activate Rho in neuropathic pain states of peripheral nerve injury. An interesting study illustrated that LPA inhibits the filopodia of growth cones [72]. LPA could be involved in C-fiber retraction, which is a hypothesis supporting functional changes induced by neuropathic pain. Together, these findings present LPA as an attractive signaling molecule in the development of neuropathic pain.

Indeed, a single i.t. injection of LPA produced marked mechanical allodynia and thermal hyperalgesia that persisted for at least 7–8 days before returning to baseline levels at day 13 [73]. Sphingosine 1-phosphate (S1P), which signals through S1P receptors and shares similar signaling pathways with LPA receptors [63], did not produce mechanical allodynia after i.t. injections in mice. These findings, therefore, highlight the specificity of LPA. Further studies have shown that LPA-induced thermal hyperalgesia and mechanical allodynia could be blocked by BoTN/C3 (i.t.) and Y-27632 [73]. In addition, LPA decreased the peptidergic [35], but not non-peptidergic C-fiber responses, but markedly increased A(δ)-fiber responses in the APF test, which strongly suggests that LPA mimics partial sciatic nerve injury by causing neuropathic pain [25].

### *Ex vivo* studies of LPA-mediated demyelination

**Figure 2:**
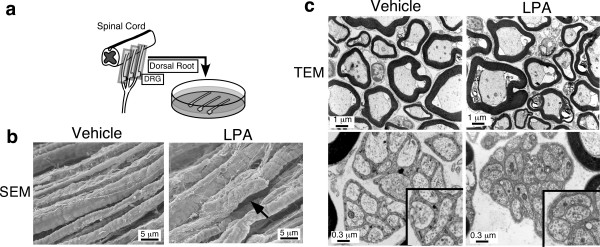
**LPA-induced demyelination of dorsal root fibers in *ex vivo *culture experiments [74].** The addition of LPA (100 nM) causes demyelination of acutely isolated dorsal root fibers after 12 h, in both scanning and transmission electron microscope (SEM and TEM) analyses. LPA also causes morphological changes in Schwann cells of the Ramaak bundle, which induce close membrane apposition.

Consistent with the fact that receptor-mediated LPA signaling influences the morphology of Schwann cells [70], the i.t. injection of LPA (1 nmol), as well as partial sciatic nerve injury, caused demyelination of the dorsal root within 24 h; this demyelination was abolished by pretreating with BoTN/C3 [73]. An *ex vivo *study using dorsal root fibers also demonstrated that the addition of LPA causes demyelination of A-fibers and damage to Schwann cells, which promotes direct contact between C fibers in the Remak bundle [74] (Fig. 2). LPA-induced demyelination was, however, reversed by BoTN/C3 or Y27632, a ROCK inhibitor. In addition, myelin basic protein (MBP) and myelin protein zero (MP0) were down-regulated. LPA-injection (i.t.) or partial sciatic nerve injury in mice produced demyelination in the dorsal root, but not in the spinal nerve, although the addition of LPA led to significant demyelination in both fibers *ex vivo*, which suggests that *in vivo *demyelination occurs specifically at the dorsal-root, proximal to the spinal cord. Furthermore, we observed that simultaneous ligation of the residual half of the sciatic nerve triggered only a slight increase in demyelination. Taken together, these findings provide speculation to the theory that certain spinal cord-originating extracellular signaling molecules, including LPA, diffuse to the dorsal root to cause demyelination.

### Involvement of LPA_1_-signaling in nerve injury-induced neuropathic pain

LPA acts through G-protein-coupled LPA receptors, designated LPA_1_, LPA_2_, LPA_3_, and LPA_4_, each exhibiting different G protein interactions [63,64]. However, only the *lpa*_1 _receptor gene is expressed in both DRG neurons and dorsal root [73]. LPA_1_-null mice reversed LPA-induced demyelination, as well as mechanical allodynia [73]. Furthermore, LPA_1 _receptor-mediated demyelination was evidenced by down-regulation of myelin-associated proteins, such as MBP and peripheral myelin protein 22 kDa (PMP22), in the dorsal root after LPA injection or peripheral nerve injury. These changes were identical to nerve injury changes resulting in neuropathic pain. Indeed, LPA_1_-null, reversed nerve injury-induced neuropathic pain and demyelination, as well down-regulation of myelin proteins and up-regulation of A-fiber Ca_v_α_2_δ-1 and spinal PKCγ [73].

### Nerve injury-induced *de novo *LPA biosynthesis

**Figure 3:**
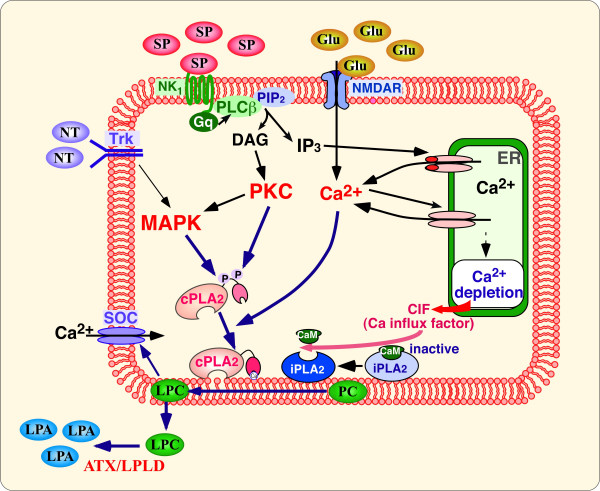
***De novo*****biosynthesis of LPA in spinalcord neurons**. The convertion of phosphatidyl choline (PC) to LPC is mediated by cPLA2 and iPLA2, which are activated by receptor-mediated MAPK, PKC, and [Ca^2+^]i increases. Autotaxin/lysophopholipase D (ATX/LPLD) subsequently converts LPC to LPA. The intense stimulation of sensory fibers might initiate *de novo *biosynthesis of LPA.

It is important to understand whether endogenous LPA plays a role in the development of neuropathic pain; if so, it will be important to demonstrate the mechanisms involved. Thermal hyperalgesia and mechanical allodynia after partial sciatic nerve ligation were mostly reversed by pretreatment with AS-ODN for LPA_1 _or in LPA_1_-null mice [73]. Because no significant nociceptive threshold change was observed in uninjured LPA_1_-null mice, it is evident that *de novo *LPA, produced by injury, is involved in the generation of a neuropathic pain state. Similar roles of *de novo*-produced LPA have also been observed in demyelination, decreased protein and gene expression of related myelin molecules (MBP and PMP22), and upregulation of PKCγ and of Ca_v_α_2_δ-1 in mice with partial sciatic nerve ligation [73]. Furthermore, LPA_1 _receptor-mediated demyelination was specific to the dorsal root after the sciatic nerve injury. Taken together, these findings suggest that LPA is biosynthesized *de novo *in the spinal cord upon intense pain signals, and is subsequently released at the dorsal root to cause demyelination. Autotaxin (or lysophospholipase D), which converts lysophosphatidyl choline (LPC) to LPA, is a key enzyme for LPA production [75]. Recent studies revealed that phosphatidyl choline is converted to LPC by cytosolic phospholipase A2 (cPLA2) or calcium-independent PLA2 (iPLA2), both of which are regulated by Ca^2+^-related mechanisms. cPLA2 is activated through membrane translocation, which is stimulated by Ca^2+ ^or phosphorylation by mitogen-activated kinase (MAPK) or PKCs [76-78], while iPLA2 is activated through the removal of calmodulin by calcium influx factor (CIF) produced after Ca^2+ ^depletion in the endoplasmic reticulum [79,80]. Therefore, intense pain-signals after nerve-injury may induce an excess release of pain transmitters, SP, and glutamate, which in turn activate both cPLA2 and iPLA2 through different pathways (Fig. 3). Neurotrophic factors (e.g., BDNF) and cytokines may also contribute to cPLA2 activation through MAPK-activating pathways. More recently, neuropathic pain was shown to be induced by LPC (i.t.) or nerve injury and was absent in LPA_1_-null or autotaxin-null mice [81,82]. Our recent findings showed that LPC did not cause demyelination in *ex vivo *experiments, although many reports have demonstrated LPC-induced demyelination *in vivo *[83-85]. Taken together, these findings might suggest that *de novo*-produced LPC in the spinal cord is transported to the dorsal root, where it is then converted to LPA.

### LPA as an initiator of nerve-injury-induced neuropathic pain

Neuropathic pain behavior at day 14 was absent after pretreatment with AS-ODN for LPA_1_, but not with later treatments that started at day 7 post-injury [73]. Neuropathic pain was also blocked when BoTN/C3 was administered within 1 h post-injury. A time-course study using Ki-16425, a short-lived LPA_1 _antagonist [86] revealed that LPA_1 _receptor signaling was found to terminate within 3 h following injury (Lin and Ueda *et al.*, unpublished data). These findings suggest that *de novo*-produced LPA after nerve injury might initiate various mechanisms of neuropathic pain through the LPA_1 _receptor [73].

### Working hypothesis for LPA_1 _signaling initiating neuropathic pain

**Figure 4:**
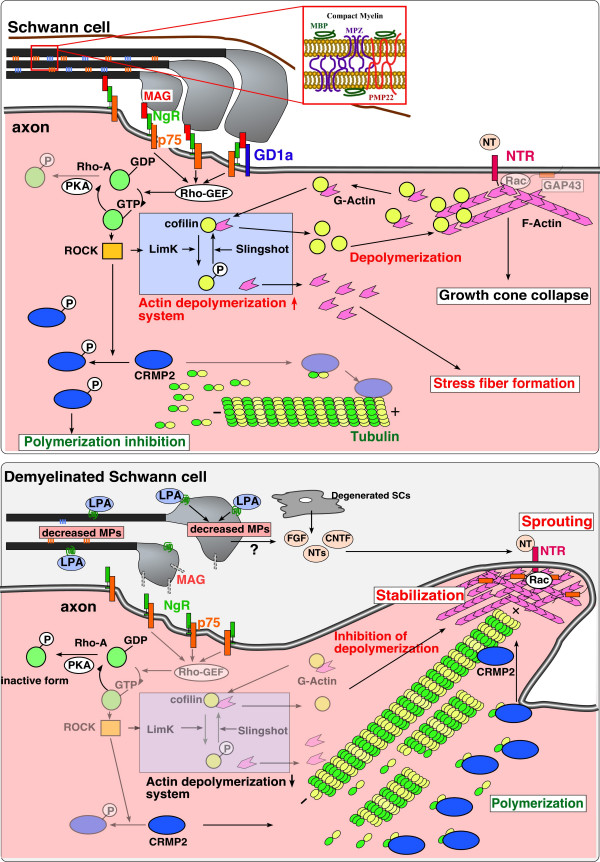
**Schematic model of LPA-induced demyelination.** The stimulation of LPA_1 _receptor first induces myelin to down-regulate compact myelin proteins, such as MBP, MPZ, and PMP22, and to loosen myelin structure. In addition, MAG is down-regulated and NOGO/p75 receptor complex (NgR/p75)-mediated activation of Rho-ROCK system is terminated. The latter mechanism results in inhibition of actin depolymerization, or sprouting. Degenerated Schwann cells (SCs) release neurotrophins, which in turn accelerate sprouting.

LPA_1 _receptor-mediated demyelination is an important subject in the field of neuropathic pain. We obtained evidence that LPA_1 _receptor activation mediates down-regulation of myelin proteins, such as peripheral myelin protein PMP22, myelin basic protein MBP, and myelin protein zero MP0, in *in vivo *injury models and *ex vivo *culture models [73,74]. Nerve injury-induced down-regulation of myelin proteins and their genes was reversed with BoTN/C3 pretreatment; however, further downstream mechanisms remain to be determined. Because the time-course of down-regulated protein levels is similar to the gene expression [74], the former mechanism seems to be a result of rapid degradation, and not a secondary event subsequent to the down-regulation of gene expression. It should be noted that myelin-associated glycoprotein (MAG) was also down-regulated, while growth associated protein 43, a marker protein for sprouting axonal growth, was up-regulated (Fujita, Ueda *et al.*, unpublished data). This is consistent with the fact that MAG inhibits axonal growth through activation of the NOGO/p75 receptor complex and leads to inhibition of actin polymerization mechanisms [87-89] (Fig. 4). Thus, it is interesting to speculate that these mechanisms are responsible for ephaptic or physical crosstalk between different modalities of fibers through sprouted A-fiber branches.

**Figure 5:**
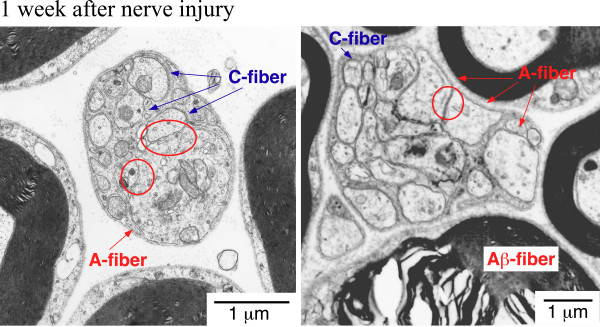
**Representative photograph of close membrane apposition between C- and A-fibers and between A-fibers. **Dorsal root was isolated from the mouse 1 week after partial sciatic nerve injury and was used for TEM analysis (Fujita and Ueda *et al.*, unpublished data).

**Figure 6:**
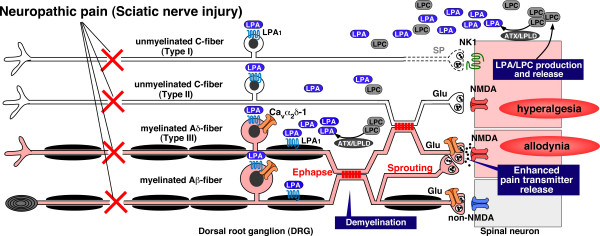
**Working hypothesis of neuropathic hyperalgesia and allodynia. **This model depicts possible mechanisms of neuropathic pain following partial sciatic nerve injury in mice. Detailed interpretation is described in the text.

In patients and experimental animals with neuropathic pain, mild tactile stimulation causes burning pain. This phenomenon, termed allodynia, has been the focus in our study of neuropathic pain mechanisms. The possibility that tactile Aβ and nociceptive Aδ or C fibers cross at the level of sensory fibers, or at the level of pain transmission in the spinal dorsal horn, would seem to be a reasonable explanation [3]. Collateral sproutings from primary afferent fibers, which induces ephaptic or physical crosstalk between different types of fibers, have long been speculated to be involved in the plasticity or reorganization mechanisms of spinal neuronal circuits for pain transmission [90]. Evidence of LPA-induced demyelination accompanying loss of insulation could account for the physical crosstalk (presumed structural basis of ephaptic crosstalk) between non-nociceptive (Aβ) and nociceptive (C and Aδ) fibers, which is observed in mice with nerve injury (Fig. 5). On the other hand, subsequent to peripheral nerve injury, the regenerating axon terminals are known to sprout to the skin area that is typically denervated [51]. Local NGF release from skin cells is expected to drive this sprouting, because anti-NGF treatment prevents sprouting [91]. Further studies focused on growth factor-induced sprouting after LPA activation are needed. Altogether, we propose a hypothesis for mechanisms of neuropathic hyperalgesia and allodynia after partial sciatic nerve injury (Fig. 6) as follows: 1) intense stimulation of sensory neurons after sciatic nerve injury activates target neurons in the dorsal horn to induce *de novo *biosynthesis of LPC, which is in turn converted to LPA by ATX/LPLD; 2) LPA is then produced at the dorsal root fibers proximal to the spinal cord. LPC may be also produced at the dorsal root, where it is converted to LPA; 3) LPA binds to LPA_1 _receptors, resulting in retraction of peptidergic unmyelinated C-fibers. The C-fibers are deprived of spinal pain transmission, due to down-regulation of various pain-related molecules (B2-type BK receptor on fibers, SP level in the dorsal horn), as well as the possible retraction of central nerve endings; 4) myelinated Aδ-fibers exhibit hypersensitivity due to up-regulation of TRPV1, B1-type BK receptor, and Ca_v_α_2_δ-1. However, it remains to be shown that the LPA_1 _receptor is involved in expression changes of key molecules. It also remains to be determined whether LPA_1 _receptor signaling directly causes the down-regulation of these molecules; 5) LPA, which is released at the dorsal root, demyelinates the Aδ- and Aβ-fibers on the dorsal root through the LPA_1 _receptor, followed by physical (or ephaptic) crosstalk between the C-fiber and Aδ-fiber, and between the Aδ-fiber and Aβ-fiber. Sprouting also causes novel pain transmission in the spinal dorsal horn. These two events following demyelination may regulate the mechanisms of neuropathic allodynia.

## Future direction

Neuropathic pain is thought to become worse with time, if not appropriately treated. This present review proposes initial mechanisms at the level of peripheral nerves following nerve injury. The development of agents to block enhanced pain transmission is an important therapeutic direction for research. It would be important to target the crosstalk between noxious and innocuous fibers after demyelination to cure or prevent neuropathic pain. Sustained mechanisms could occur at the spinal and supraspinal levels. Plasticity or reorganization of neural networks in pain and pain-inhibitory pathways could make treatment more problematic. Pain induces pain in a vicious circle. Complete blockade of the plasticity responsible for neuropathic pain at the initial stage and at the peripheral level might be an appropriate strategy.
